# Concentration of Selected Elements in the Infrapatellar Fat Pad of Patients with a History of Total Knee Arthroplasty

**DOI:** 10.3390/ijerph16101734

**Published:** 2019-05-16

**Authors:** Żaneta Ciosek, Danuta Kosik-Bogacka, Natalia Łanocha-Arendarczyk, Karolina Kot, Maciej Karaczun, Paweł Ziętek, Patrycja Kupnicka, Aleksandra Szylińska, Iwona Rotter

**Affiliations:** 1 Departament of Biology and Medical Parasitology, Pomeranian Medical University in Szczecin, Powstańców Wielkopolskich 72, 70-111 Szczecin, Poland; nlanocha@pum.edu.pl (N.Ł.-A.); kotkarolina17@gmail.com (K.K.); 2 Department of Medical Rehabilitation and Clinical Physiotherapy, Pomeranian Medical University in Szczecin, Żołnierska 54, 71-210 Szczecin, Poland; aleksandra.szylinska@gmail.com (A.S.); iwrot@wp.pl (I.R.); 3 Independent of Pharmaceutical Botany, Department of Biology and Medical Parasitology, Pomeranian Medical University in Szczecin, Powstańców Wielkopolskich 72, 70-111 Szczecin, Poland; kodan@pum.edu.pl; 4 Chair and Clinic of Orthopaedics, Traumatology and Oncology, Pomeranian Medical University, Unii Lubelskiej 1,71-252 Szczecin, Poland; karaczun@orthomail.com (M.K.); paulz@wp.pl (P.Z.); 5 Department of Biochemistry and Medical Chemistry, Pomeranian Medical University, Powstańców Wielkopolskich 72, 70-111 Szczecin, Poland; pkupnicka@gmail.com

**Keywords:** infrapatellar fat pad, total knee replacement, biological factors, environmental factors

## Abstract

In the period of long development, the human body adapted to specific concentrations of trace elements. Any changes in the trace element content manifesting in a deficiency or excess of metals in the human body may impair the functioning of the skeletal and articular system and of the organs, and may also predispose the body to the development of diseases, i.e., osteoporosis. Our study aimed to analyze the concentrations of calcium (Ca), magnesium (Mg), fluorides (F-), and lead (Pb) in the infrapatellar fat pad (Hoffa’s fat pad) of subjects who had undergone a total knee replacement (TKR) surgery. We also endeavored to establish how concentration levels of those elements are affected by selected biological and environmental factors. The studied group comprised 48 residents of Western Pomerania Province: 34 women (n = 34) aged 56–87 and 12 men (n = 12) aged 59–85. Concentration levels of Ca, Mg, and Pb were established using inductively coupled plasma-atomic emission spectrometry (ICP-AES). A Thermo Orion ion-selective electrode was applied for measuring F- concentration. Subjects aged 75–87 showed higher Mg concentration values than those aged 56–74. Big city residents and smokers were found to have higher infrapatellar fat pad Mg concentration than their non-smoking counterparts and small town dwellers. Of all the elements whose concentrations we analyzed in our studies, only magnesium was found to correlate with smoking, place of residence, and age. Our findings regarding the quantities of selected elements in the infrapatellar fat pad may be used for the interpretation and analysis of biological, morphological, and mechanical changes in the human body.

## 1. Introduction

The infrapatellar fat pad or the Hoffa’s fat pad (Lat. *Corpus adiposum infrapatellare*, HFB) is a component of the knee joint. It is located between the fibrous membrane of the joint capsule formed by the quadriceps muscle tendon and the synovial membrane. Its physiological function remains unknown. Some researchers believe it may serve a protective function, be involved in biomechanics of the joint, or act as storage for reparative cells after injury [1]. The infrapatellar fat pad is the site of production of growth factors, adipocytokines, as well as proinflammatory cytokines and complement components, all of which may affect the metabolism of the synovium and cartilage [2,3,4,5].

Calcium (Ca), a microelement essential for the proper functioning of the body, regulates a range of intracellular and extracellular processes. Together with magnesium (Mg), it guarantees adequate mineralization and growth of the bone tissue. The average adult human body contains approximately 1200 g of calcium, which corresponds to 2% of the total body mass. Magnesium stimulates the activity of osteoblasts and phosphatase enzymes, which play an active role in osteogenesis [6]. The human body of a 70 kg person contains between 20 and 35 g of magnesium [7]. The element is present in bones, muscles, soft tissue, and intercellular fluids, making up 60%, 30%, 9%, and 1% of their chemical composition, respectively [8].

The fluoride ion (F-) plays a significant biological role in the human body, participating in the binding of magnesium, calcium, phosphorus, and in the mineralization of hard tissue. The concentration of F- in soft tissues does not exceed 1 mg per 1 kg^−1^ of body mass dm, which is comparable to the concentration of fluorides in the plasma [9]. Lead (Pb), a toxic heavy metal, is involved in the metabolism of vitamin D by lowering the concentration of its active form. The metal has also been recognized as one of the factors contributing to the development of osteoporosis, particularly in women during perimenopause [10,11]. The lead that enters the human body comes through inhalation, ingestion, or is absorbed through the skin. The rate of lead absorption depends on the form it occurs in, route of absorption, time of exposure, age, and sex [12].

Little research has been dedicated to the analysis of the mineral composition of the infrapatellar fat pad, in particular, the correlation between the concentrations of particular elements in periarticular structures in patients afflicted with osteoarthritis of the knee [13]. Therefore, it was a reasonable decision to investigate the Hoffa’s fat pads levels of Ca, Mg, F-, and Pb in patients who had undergone total knee replacement (TKR) and had a history of gonarthrosis. The study incorporated an analysis of the influence of selected environmental factors, such as the place of residence, smoking, and nutritional status, expressed in terms of BMI (Body Mass Index) on the concentration of some elements in the infrapatellar fat pad.

## 2. Materials and Methods

Ethical approval for this study was obtained from the Bioethics Commission of the Pomeranian Medical University in Szczecin (resolution no. KB-0012/56/14). Prior to conducting research, each of the patients obtained a full explanation of the characteristics and purpose of the study and provided written consent to participate in the study. A cohort of 46 patients, residents of the Western Pomerania region, Poland, aged 56–87 (mean age 73.4 ± 8.5 years), were included in the study. 34 were females aged 56–87 (mean age 73.4 ± 8.5 years) and the remaining 12 were males aged 56–85 (mean age 73.1 ± 8.4 years). The patients were hospitalized in the Department and Clinic of Orthopedics, Traumatology and Oncology of the Motor System at the Pomeranian Medical University in Szczecin. All patients underwent knee joint replacement with reconstruction of the intercondylar area, using an autologous bone graft. The main indication for the procedure was degeneration of either the left or the right knee joint (26 and 20 patients respectively), resulting from an underlying disease, i.e., psoriatic arthritis (n = 1) or knee joint injury (n = 1).

Prior to surgery, relevant information was obtained from each patient regarding their health status, environmental exposure (i.e., place of residence), and smoking status. Subsequently, based on their BMI scores, the patients were divided into two groups: One with BMI scores <30 and one with scores ≥30 kg/m^2^.

The patients were also divided into two age groups: 56–74 years old (HS1) and 75–87 years old (HS2). Pre-operative procedures that the subjects underwent included diagnostic investigations, such as an X-ray and/or MRI scan of the knee joint. None of the patients suffered any post-operative complications. A sample of the infrapatellar fat pad tissue was collected from each patient during the procedure (n = 46). In all cases, the samples were classified as medical waste and disposed of. The material was harvested in sterile conditions. The collected samples were labeled with code numbers and stored in sterile containers at −27 °C.

The material was weighed with a high accuracy analytical balance Sartorius ENTRIS124-1S Analytical Balance 120 g × 0.1 mg (Sartorius AG, Goettingen, Germany) to an accuracy of 0.0001 g, and then dried to constant weight at 105 °C. The dried material was subsequently crushed in an agate mortar and subjected to microwave mineralization using the MARS 5 CEM system.

Ca, Mg, and Pb concentrations were determined using atomic emission spectrometry with excitation in inductively coupled plasma (ICP-AES, ICAP 7400 Duo, Thermo Scientific, Waltham, MA, USA), while a Thermo Orion I (Thermo Scientific) ion-selective electrode was applied for measuring the concentration of F-.

Statistical analysis was performed using Statistica 10.0 software (StatSoft, Inc. Tulsa, OK, USA). The analyses included determination of mean concentration levels of selected elements (Ca, Mg, F-, and Pb) in the infrapatellar fat pad tissue. Distribution of Ca, Mg, F-, and Pb concentrations was tested for normality by the Shapiro–Wilk test. Arithmetic mean (AM), standard deviation (SD), median (med.), and range (min–max) were used for presentation of the findings. The correlation between concentration levels of selected elements in the infrapatellar fat pad and factors like age, sex, place of residence, BMI scores, and smoking was established using the Mann–Whitney U test. Spearman’s rank correlation coefficient was applied for analysis of the relationship between particular elements. The level of significance was set at *p* ≤ 0.05.

## 3. Results

### 3.1. Characteristics of the Entire Cohort

Mean concentrations of Ca, Mg, F-, and Pb in the infrapatellar fat pad of the studied patients were 1290.38, 46.57, 89.17, and 0.51 mg·kg^−1^ dry mass respectively (Table 1).

### 3.2. Concentrations of Ca, Mg, F-, and Pb in the Infrapatellar Fat Pad in Relation to Selected Biological and Environmental Factors

The analysis of Mg levels revealed their statistically significant correlations with age (*p* = 0.012), place of residence (*p* = 0.047), and smoking (*p* = 0.038). No other statistically significant correlations were observed (Table 2).

### 3.3. Analysis of the Relationship between the Concentration Levels of Selected Elements in the Infrapatellar Fat Pad and Selected Biological and Environmental Factors

A considerable increase in the concentration of magnesium was observed in subjects aged over 75 years (r = 0.378, *p* = 0.012), and those residing in towns and villages with populations below 100,000 inhabitants (r = −0.298, *p* = 0.047). Compared to their non-smoking counterparts, smokers were found to have lower levels of the infrapatellar fat pad magnesium (r = −0.312, *p* = 0.038). No other statistically significant relationship was observed (Table 3).

## 4. Discussion

All organisms are made of chemical elements. The quality of human life is closely related to the chemical composition of the natural environment and food. In the period of long development, the human body adapted to specific concentrations of trace elements. Any changes in the trace element content, manifesting in a deficiency or excess of metals in the human body, may impair the functioning of the skeletal and articular system and the organs, and may also predispose the body to the development of diseases, i.e., osteoporosis.

Concentration levels of chemical elements in various types of human bones are being investigated by a good number of research centers. Most of these studies focus on the chemical makeup of the human femur and ribs. Similar analyses of particular elements are carried out in full blood, blood plasma, serum, urine, or horny derivatives of the epidermis. The chemical composition of the infrapatellar fat pad in patients with osteoarthritis has scarcely been covered in existing literature [13]. The few studies on the subject found that the mean concentration levels of F- in the infrapatellar fat pad is 62.14 mg∙kg^−1^ dm (dry mass) [14].

Based on the results of other studies, the mean concentration of Ca in the knee joint components usually varies between 3000 and 5050 mg∙kg^−1^ dm [15,16], whereas its concentration in the infrapatellar fat pad was lower than the relevant Ca levels in the anterior cruciate ligament and the meniscus, where it amounts to 1290.38 mg∙kg^−1^ dm. The discrepancy between the figures may be due to structural inhomogeneity of these components.

Mean level of Mg in the knee joint varies from 78 to 1600 mg∙kg^−1^ dm [15,16]. Our study reports the infrapatellar fat pad concentration of the element at the level of 46.57 mg∙kg^−1^ dm, much lower than its concentration in the bone tissue, which, because of the longer time it needs to form, may reflect chronic exposure and provide a basis for indirect assessment of the extent of environmental exposure.

The analysis of F- levels in bone structures demonstrated that its highest concentration is in the vertebrae (>500 mg∙kg^−1^ dm), ribs (100-500 mg∙kg^−1^ dm), and the femur (100-450 mg∙kg^−1^ dm), whereas its concentration in the tibia varies between 560 and 635 mg∙kg^−1^ dm [17]. In their study carried out on a cohort of 20 patients (residents of the Western Pomerania region in 2017), Kot et al. [14] established the mean concentration of F- in the infrapatellar fat pad at the level of 62.14 mg∙kg^−1^ dm, although, in two females, the level exceeded 1000 mg∙kg^−1^ dm. One of these patients also had high levels of F- in two other parts of the knee joint, while in the second patient, the F- levels in the other parts of the knee joint were below 550 mg∙kg^−1^ dm. The mean concentration value of F- in the infrapatellar fat pad in the Western Pomerania residents reported in our study is 89.17 mg∙kg^−1^ dm. Any differences between obtained values might result from the size and diversity of the studied samples.

Mean concentration of Pb in the knee joint elements vary between 0.20–4 mg∙kg^−1^ dm [16,17]. Our study has shown that the mean concentration of this element in the infrapatellar fat pad is lower than in the bone tissue, and amounts to 0.51 mg∙kg^−1^ dm. Bone is the major reservoir of body lead storage. The predominant mean half-life of this toxic metal in bone tissue is 20–30 years [18]. Its accumulation in bones begins during prenatal development and continues throughout life. The rate of lead absorption is determined by the extent of exposure.

A number of studies have investigated the relationship between age and concentration levels of selected elements, in particular tissues and organs. Zaichick and Zaichick [19] found that Pb levels in the femur of subjects aged 15–35 years were higher than in those aged 36–55 years. Analyzing the concentration of Ca in the spongy head of the femur, Brodziak-Dopierała et al. [20] observed that its levels decreased with age from 149 to 101 mg/g dm. A similar pattern was observed in the cortical and spongy bone of the intertrochanteric line area of the hip joint [21].

A considerable decrease has been observed in the levels of intracellular free magnesium in healthy individuals aged over 65 years [22,23]. Zaichick and Zaichick [19] recorded higher Mg levels in femoral samples collected from 15- to 35-year-old individuals than in those harvested from older subjects aged 36–55 years (2009 and 1687 mg∙kg^−1^ dm, respectively). An inverse relationship was observed by Brodziak-Dopierała et al. [20] in patients suffering from osteoarthritis and those undergoing hip replacement surgery following hip fracture. The authors demonstrated that bone Mg levels in patients aged 59 were lower than in those over 80 years old. Our study has shown that bone Mg levels of subjects aged between 75–87 years were significantly higher than in their younger counterparts aged 56–74. Analyzing the levels of F- in the femur of women from two age groups (>65 and <65 years old), Palczewska-Komsa et al. [24] found that the former had a considerably higher concentration of the element than the latter one (525 and 275 mg∙kg^−1^ dm, respectively). In males, the concentration of fluorides in the femur showed an opposite pattern—those less than 65 years old showed higher F- levels than their older counterparts (552 and 268 mg∙kg^−1^ dm, respectively). Łanocha-Arendarczyk et al. [17] found that the concentration levels of F- in the spongy bone of the femur were 1.5 times higher in subjects aged over 60 years old than in those less than 65 years old. (519.46 and 350.39 mg∙kg^−1^ dm, respectively) The pattern, however, was not observed in the compact part of the bone (455.7 and 412.93 mg kg^−1^ dm, respectively). Studies into the concentration levels of heavy metals in bones showed that Pb levels increase with age. Kuo et al. [25] observed that the concentration of lead in the hip joint in Taiwanese patients rose with age. Subjects aged 41–60, 61–80, and over 80 years showed an increasing trend in Pb concentrations: 6.13, 7.62, and 9.36 mg∙kg^−1^, respectively.

Analyzing hip joint bone samples harvested from a number of subjects of various age groups populating the Upper Silesian Industrial Region, Brodziak-Dopierała et al. [21] recorded statistically significant differences in the cartilage concentration of Pb between those aged 50–60 years and individuals aged 71–80 years. Similar differences were observed in the compact bone concentrations of Pb between groups aged 50−60 years and 61−70 years.

A nationwide study by Jarosz and Respondek [26] demonstrated that nearly 70% of Polish men and 90% of women aged over 18 years suffered from dietary Ca deficiency. Analyzing differences between the chemical composition of the head of the femur samples collected from subjects aged 60–82 (inhabitants of the Upper Silesian Region) found that the levels of Ca were significantly higher in males (29099.37 mg∙kg^−1^ dm) than in females (28515.42 mg∙kg^−1^ dm) [27]. In their study, Tohno et al. [28] recorded that Ca concentration measured in the anterior cruciate ligament was significantly higher in males than in females (4030 mg∙kg^−1^ dm and 3390 mg∙kg^−1^ dm, respectively). Ziola-Frankowska [29] did not find differences in Ca concentration between men and women in the parts of the knee joint taken from patients from the Silesia region. Investigating the chemical makeup of the femur head samples collected from individuals populating the Upper Silesian Region, Brodziak-Dopierała et al. [27] noticed a higher concentration of Mg in males (1818.91 mg∙kg^−1^ dm) compared to the relevant values recorded in women (1352.49 mg∙kg^−1^ dm).

Palczewska-Komsa et al. [30] observed statistically significant differences between the femur concentrations of F- in men and women. The latter group showed a nearly 50% higher concentration of the element. Similarly, Kot et al. [14] recorded approximately 1.5 times higher F- concentration in cartilage samples collected from females than in those collected from males (661.96 and 278.58 mg∙kg^−1^ dm). The concentration levels of F- in spongy bone sampled from females were twice as high as in males (514.84 and 22537 mg∙kg^−1^ dm, respectively). Concentration of F- recorded in the anterior cruciate ligament tissue was also higher in women than in men and equaled respectively 125.99 and 71.90 mg∙kg^−1^ dm. Studies by Brodziak-Dopierała et al. [31] and Yoshinaga et al. [32] showed higher femur Pb concentrations in men than in women. The findings were confirmed by Garcia et al. [33] and Zioła-Frankowska et al. [29].

The role of Ca in the regulation of metabolism and body mass in humans and laboratory animals has been reported by a number of research publications. Loos et al. [34] observed that women with low Ca intake displayed the highest body fat percentage. Kamyecheva et al. [35] found a relationship between the consumption of Ca and BMI values in males, although the correlation was not observed in women.

Numerous studies have demonstrated lower blood plasma Mg concentration levels in obese individuals, both children and adults [36,37,38]. Studies conducted between 2012 and 2013 on Canadian subjects aged 3–79 years showed a negative correlation between BMI scores and serum Mg concentration levels [39]. Skalnaya et al. [40] found that Pb concentration in male and female hair was higher in subjects with increased BMI, regardless of age.

Our study on the interplay of selected biological and environmental parameters and the levels of some elements has not provided conclusive evidence of the influence of such factors as sex, age, BMI, and alcohol consumption on the concentrations of calcium, fluorides, or lead in the infrapatellar fat pad. What we did find was a statistically significant correlation between the concentration of magnesium and age, place of residence, and smoking.

In Europe, there are insufficient amounts of comparative data concerning concentration of selected elements in the infrapatellar fat pad. It is therefore reasonable to carry out relevant analyses in post-TKR patients with a history of gonarthrosis. Studies investigating the relationship between concentration levels of some elements and place of residence have been mainly carried out on bone tissue. Zioła-Frankowska et al. [29] did not find any material differences between Ca and Mg concentrations in compact or spongy bone samples of the femur, collected from small town inhabitants and city dwellers. Palczewska-Komsa et al. [30] found no difference in the femur concentrations of F- between subjects residing in Szczecin and those living in villages or other towns with populations of 6000–70,000 people. This study has found that Mg levels in the infrapatellar fat pad of big city dwellers are significantly lower than those recorded in the inhabitants of small towns and villages (70.61 and 39.02 mg/kg dm, respectively). The difference may result from the fact that the number of the participants living in big cities of over 100,000 inhabitants was three times bigger than the relevant number of smaller city and village dwellers.

According to WHO estimates (2017), there are approximately one billion smokers worldwide. An estimated 40% of Poles smoked cigarettes in the 1980s, compared to around 20% recorded in 2015 [39]. Nicotine has been found to adversely affect the proliferation of osteoblasts and fibroblasts, as well as the production of collagen [31,41,42,43].

Smoking women who have entered perimenopause show a lower level of osteocalcin [42,44]. Individuals who smoke a minimum of 20 cigarettes a day demonstrate impaired intestinal Ca absorption [45]. In view of the above findings, tobacco smoking was recognized as a risk factor for osteoporosis and consequential bone fractures [46,47]. Little is known, though, about the influence that smoking tobacco has on mineral balance of Mg [48]. Some studies report lower levels of Mg in the serum of smokers [49,50]. This study has established that the concentration of magnesium in the infrapatellar fat pad of tobacco smokers is considerably lower than in non-smoking individuals (less than 26 and 51 mg∙kg^−1^ dm, respectively).

Of all the elements we have analyzed, only Mg concentrations showed different levels in smokers and non-smokers, subjects living in big cities of over 100,000 and those residing in smaller towns and villages, as well as between subjects aged 56–74 years old and those aged 75–87 years old. Higher Mg levels were observed in the subjects aged 75–87 years, inhabitants of smaller towns and villages, and non-smokers.

## 5. Conclusions

The data our study has provided on the concentration levels of selected elements in the infrapatellar fat pad can be used for the interpretation and analysis of biochemical, morphological, and mechanical changes taking place in the human body.

## 6. Limitations

This study has potential limitations. First of all, the sample size was relatively small, which makes it difficult to identify significant relationships from the data. Secondly, all relevant tests and investigations were carried out in one centre and the subjects involved were all residents of a limited area of Western Pomerania, Poland. Another limitation results from the fact that the findings that this research yielded concerning the chemical make-up of the infrapatellar fat pad could not be verified by reference to the results of relevant studies carried out by other authors.

## Figures and Tables

**Table 1:** Characteristics of the entire cohort.

Elements	AM	SD	Min	Max	Me
Ca	1290.38	1720.01	129.90	8297.79	565.05
Mg	46.57	35.69	12.81	188.43	33.25
F-	89.17	49.28	20.49	186.95	83.34
Pb	0.51	0.23	0.00	0.84	0.54

Legend: AM—arithmetic mean, SD—standard deviation; Me—median; min—minimum, max—maximum, Ca—calcium, Mg—magnesium, F^−^—fluorine, Pb—lead. Notes: Concentration of the elements is expressed as mg∙kg-1 dry mass.

**Table 2:** Concentration levels of Ca, Mg, F-, Pb in the infrapatellar fat pad and their relation to selected biological and environmental factors.

	**Gender**						
	**M (n = 12)**			**F (n = 34)**			***p***
	**AM ± SD**	**Me**	**Min–Max**	**AM ± SD**	**Me**	**Min–Max**	
Ca	1329.90 ± 2271.86	509.73	180.25–8297.79	1276.43 ± 1520.86	613.05	129.90–6705.32	0.851
Mg	60.0 ± 55.83	34.7	12.81–188.43	41.83 ± 24.67	33.25	16.89–112.79	0.755
Pb	0.46 ± 0.27	0.47	0.001–0.828	0.54 ± 0.21	0.61	0.09–0.84	0.402
F-	104.54 ± 54.16	81.05	52.95–186.95	85.33 ± 48.45	83.34	20.49–182.35	0.337
	**Age**						
	**HS1 (n = 26)**			**HS2 (n = 20)**			***p***
	**AM ± SD**	**Me**	**Min–Max**	**AM ± SD**	**Me**	**Min–Max**	
Ca	1201.77 ± 1501.48	592.21	171–6705.32	1405.56 ± 2003.94	559.18	129.90–8297.79	0.991
Mg	37.06 ± 27.69	28.52	12.81–136.69	58.94 ± 41.49	50.49	17.39–188.43	0.012
Pb	0.54 ± 0,21	0.57	0.04–0.84	0.48 ± 0.25	0.51	0.001–0.83	0.445
F-	100.27 ± 48.44	105.56	24.12–186.95	72.52 ± 47.66	60.3	20.49–182.35	0.112
	**Place of residence**						
	**Under 100,000 (n = 11)**			**Over 100,000 (n = 35)**			***p***
	**AM ± SD**	**Me**	**Min–Max**	**AM ± SD**	**Me**	**Min–Max**	
Ca	1523.87 ± 2339.66	736.15	186.75–8297.79	1216.99 ± 1510.76	544.21	129.90–6705.32	0.817
Mg	70.61 ± 53.94	58.49	12.81–188.43	39.02 ± 24.17	30.39	16.89–112.79	0.047
Pb	0.48 ± 0.28	0.61	0.001–0.83	0.52 ± 0.21	0.53	0.09–0.84	0.837
F-	83.23 ± 48.12	68.01	37.64–175.81	91.33 ± 50.63	94.67	20.49–186.95	0.796
	**BMI**						
	**<30 (n = 21)**			**≥30 (n = 25)**			***p***
	**AM ± SD**	**Me**	**Min-Max**	**AM ± SD**	**Me**	**Min–Max**	
Ca	934.63 ± 908.29	544.21	129.90–3755.05	1589.21 ± 2157.73	640.2	171.05–8297.79	0.708
Mg	43.12 ± 29.76	32.19	12.81–136.69	49.47 ± 40.38	34.31	16.89–188.43	0.860
Pb	0.51 ± 0.21	0.54	0.04–0.82	0.52 ± 0.24	0.53	0.001–0.84	0.724
F-	68.23 ± 38.32	52.95	20.49–130.96	101.29 ± 51.71	98.24	24.12–186.95	0.121
	**Smoking**						
	**NS (n = 39)**			**S (n = 7)**			***p***
	**AM ± SD**	**Me**	**Min–Max**	**AM ± SD**	**Me**	**Min–Max**	
Ca	1392.69 ± 1835.57	585.89	129.90–8297.79	720.35 ± 634.14	326.94	171.05–1779.05	0.392
Mg	50.39 ± 37.44	40.62	12.81–188.43	25.28 ± 7.20	23.38	17.63–37.98	0.038
Pb	0.51 ± 0.24	0.54	0.001–0.84	0.52 ± 0.19	0.45	0.29–0.75	0.807
F-	83.88 ± 49.54	69.81	20.49–186.95	123.52 ± 34.77	131.29	75.58–155.90	0.093

Legend: AM—arithmetic mean, SD—standard deviation; Me—median; min—minimum, max—maximum, F—females, M—males; HS 1—subjects aged 56–74 years; HS 2—subjects aged 75–87 years; BMI—body mass index, NS—non-smoking, S—smoking, Ca—calcium, Mg—magnesium, F-—fluorine, Pb—lead, n—number of subjects, *p*—statistical significance. Notes: Concentration of the elements is expressed as mg∙kg^−1^ dry mass.

**Table 3:** Analysis of the relationship between the concentration levels of selected elements in the infrapatellar fat pad and selected biological and environmental factors.

	Ca		Mg		F		Pb	
	r	*p*	r	*p*	r	*p*	r	*p*
**Płeć**	0.030	0.844	−0.048	0.749	−0.183	0.333	0.127	0.401
**Age**	0.065	0.668	0.378	0.010	−0.342	0.065	−0.154	0.306
**Place of residence**	−0.036	0.810	−0.298	0.045	0.052	0.784	0.033	0.830
**BMI**	0.006	0.969	0.010	0.946	0.218	0.247	0.056	0.711
**Smoking**	−0.130	0.389	−0.312	0.035	0.317	0.088	−0.039	0.798

Legend: Ca—calcium, Mg—magnesium, F-—fluorine, Pb—lead., BMI—body mass index, r—correlation coefficient, *p*—statistical significance.
